# MEK Inhibitor Associated Airway Injury in an Infant With Noonan Syndrome: A Case Report

**DOI:** 10.1002/lary.70749

**Published:** 2026-07-26

**Authors:** Veronica Drozdowski‐Nuccio, Rachel D. Yoon, Steven Tomasi, Dana M. Thompson, Inbal Hazkani

**Affiliations:** ^1^ Division of Pediatric Otolaryngology‐Head and Neck Surgery Ann & Robert H. Lurie Children's Hospital of Chicago Chicago Illinois USA; ^2^ Department of Otolaryngology‐Head and Neck Surgery, Feinberg School of Medicine Northwestern University Chicago Illinois USA; ^3^ Department of Biological Sciences Northwestern University Evanston Illinois USA

**Keywords:** MEK inhibitor, Noonan syndrome, severe side effects, toxicity, trametinib

## Abstract

An infant with Noonan syndrome treated with trametinib developed extensive mucosal sloughing of the upper and lower airway, followed by severe supraglottic scarring. Clinicians should consider airway toxicity as a potential adverse effect of MEK inhibitor therapy.

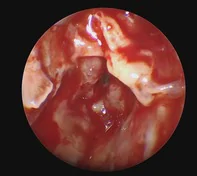

## 1 Introduction

Noonan syndrome (NS) is a relatively common autosomal dominant multisystem disorder with an estimated prevalence of 1 in 1000–2500 live births. It is characterized by variable features including distinct craniofacial morphology, congenital heart defects, short stature, and lymphatic abnormalities [1, 2, 3]. Hypertrophic cardiomyopathy (HCM) occurs in approximately 20% of individuals with NS and is associated with increased morbidity and mortality, particularly in those diagnosed within the first 6 months of life [3]. Management strategies are typically symptom‐specific and tailored to individual patients. While upper airway anomalies are uncommon in NS, some patients require invasive respiratory support due to cardiopulmonary complications [1, 2].

NS is part of the RASopathy spectrum—disorders caused by germline mutations in the RAS/MAPK signaling pathway, which regulates cell proliferation and differentiation [1, 3, 4, 5]. MEK inhibitors, such as trametinib, have emerged as potential targeted therapies in RASopathies. Though originally developed for oncologic use, early clinical case reports suggest that these agents may improve or even reverse cardiac and lymphatic manifestations in NS. However, their use has also been associated with notable side effects, including mild to moderate skin toxicities, rhabdomyolysis, and gastrointestinal or cardiac complications [4, 5].

We present a case of a 2‐month‐old infant with NS due to a KRAS mutation and severe early‐onset HCM requiring mechanical ventilation. The patient received trametinib, with subsequent development of mucosal injury, tracheostomy‐associated complications, and extensive airway scarring. This case highlights an underappreciated adverse‐effect profile of trametinib and underscores the need for multidisciplinary management when initiating MEK inhibition in young patients with NS.

## 2 Case Report

A 2‐month‐old infant with genetically confirmed NS and severe HCM was referred to the otolaryngology service for airway evaluation due to failure to wean from mechanical ventilation. The patient was born at 37 weeks' gestation, with prenatal findings of cystic hygroma, cranial ventriculomegaly, and renal anomalies. Postnatally, a KRAS mutation consistent with a severe NS phenotype was identified. Their clinical course was complicated by progressive HCM with extrinsic tracheal compression.

Following multidisciplinary discussion, trametinib was initiated as targeted therapy for cardiomyopathy at 0.015 mg/kg/day and gradually titrated over 57 days to a maximum total daily dose of 0.1 mg/day (approximately 0.017 mg/kg/day based on patient weight at the time).

Initial microdirect laryngoscopy and bronchoscopy (MLB) demonstrated bilateral true vocal fold inflammation, posterior glottic erosion, granulation tissue, and ovoid mid‐tracheal compression (Figure 1). Granulation tissue was removed, and the patient was extubated to noninvasive support; however, recurrent respiratory distress necessitated reintubation several days later. Repeat flexible laryngoscopy after development of oral cavity bleeding revealed glottic edema, mucosal sloughing, and persistent bleeding around the endotracheal tube, prompting concern for mucosal fragility. Although clear evidence of systemic mucositis involving the gastrointestinal tract or eyes was not identified, the patient developed several additional mucocutaneous abnormalities during therapy, including significant skin breakdown of the right nare, pressure injury associated with the BiPAP interface and upper lip, and episodes of voluminous bloody oral secretions with expectoration of tissue fragments. While these findings may have been multifactorial in the setting of prolonged respiratory support and critical illness, they raised additional concern for impaired mucosal and skin integrity during trametinib therapy.

**FIGURE 1:**
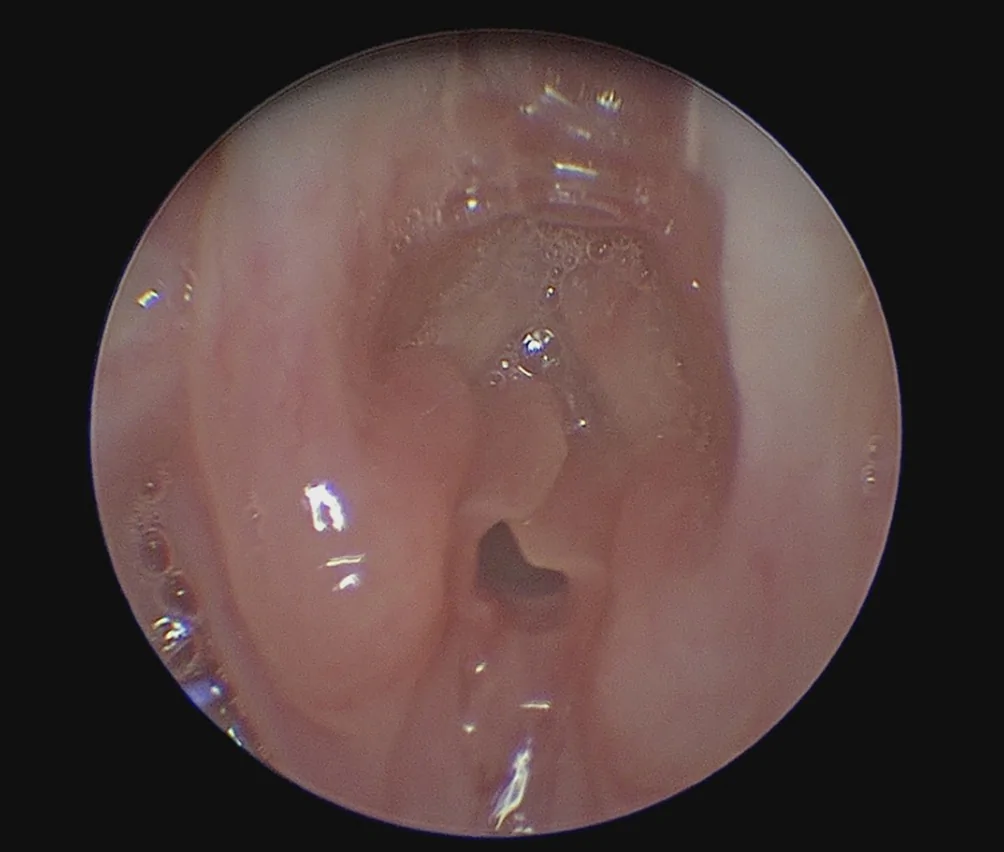
Initial microdirect laryngoscopy and bronchoscopy demonstrating glottic edema and granulation tissue, and distal airway narrowing. [Color figure can be viewed in the online issue, which is available at www.laryngoscope.com]

Given the risk of airway loss in case of accidental extubation and in the setting of prolonged intubation, the patient underwent tracheostomy. Intraoperative MLB revealed diffuse mucosal sloughing involving the oropharynx, supraglottis, glottis, subglottis, trachea, and bilateral mainstem bronchi (Figure 2). Postoperatively, the patient developed severe peristomal skin breakdown (Figure 3) and pressure injury to the posterior neck from tracheostomy ties. During routine care, they experienced accidental decannulation with the creation of a false tract prior to successful recannulation.

**FIGURE 2:**
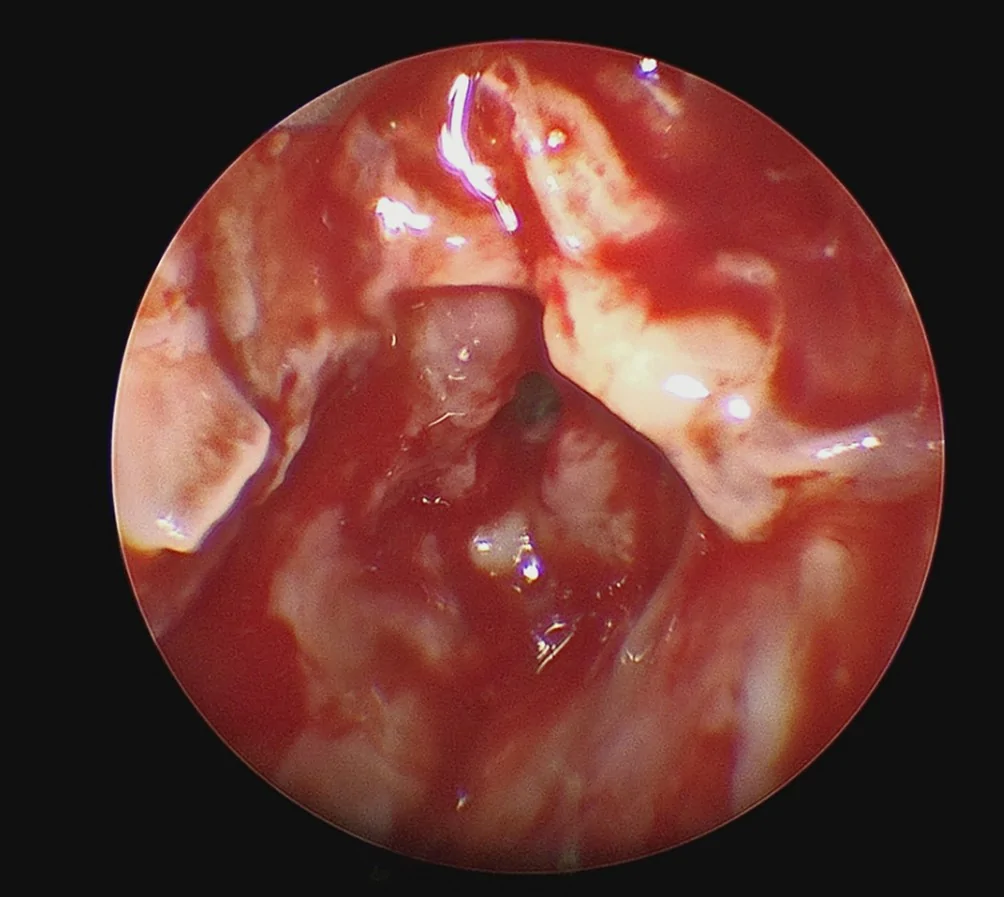
Intraoperative microdirect laryngoscopy and bronchoscopy revealed diffuse mucosal sloughing involving the supraglottis, glottis, subglottis, trachea, and bilateral mainstem bronchi. [Color figure can be viewed in the online issue, which is available at www.laryngoscope.com]

**FIGURE 3:**
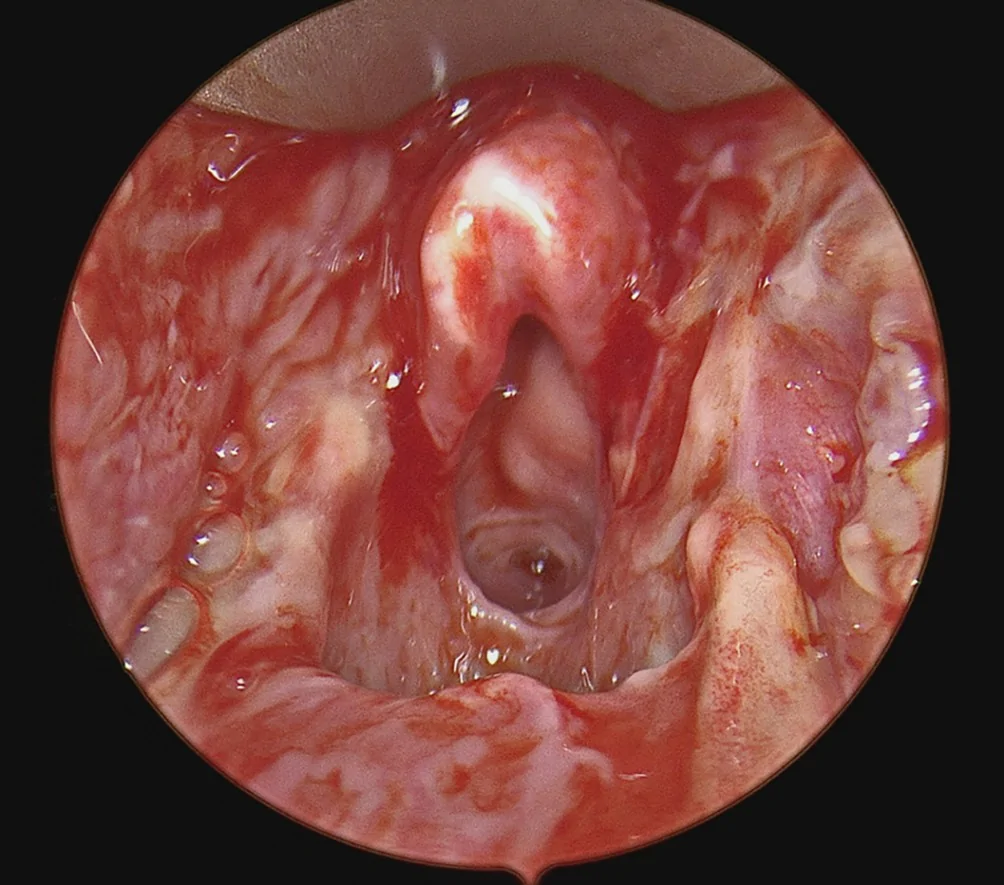
Peristomal skin breakdown. [Color figure can be viewed in the online issue, which is available at www.laryngoscope.com]

Following this event, a shared decision was made to discontinue trametinib due to the severity of mucosal injury, timing of symptom onset, and concern for drug‐induced toxicity.

A multidisciplinary team, including otolaryngology, neonatology, and wound care, managed the stomal breakdown conservatively with serial dressing changes, resulting in gradual healing. Surveillance MLB 4 weeks posttracheostomy demonstrated interval improvement in mucosal integrity; however, significant supraglottic scarring was noted, including aryepiglottic fold adhesion to the posterior pharyngeal wall, lateral pharyngeal wall tethering, and narrowing of the esophageal inlet. Subglottic stenosis with fibrous exudate caused critical airway narrowing, and persistent edema led to crowding of the true vocal folds (Figure 4).

**FIGURE 4:**
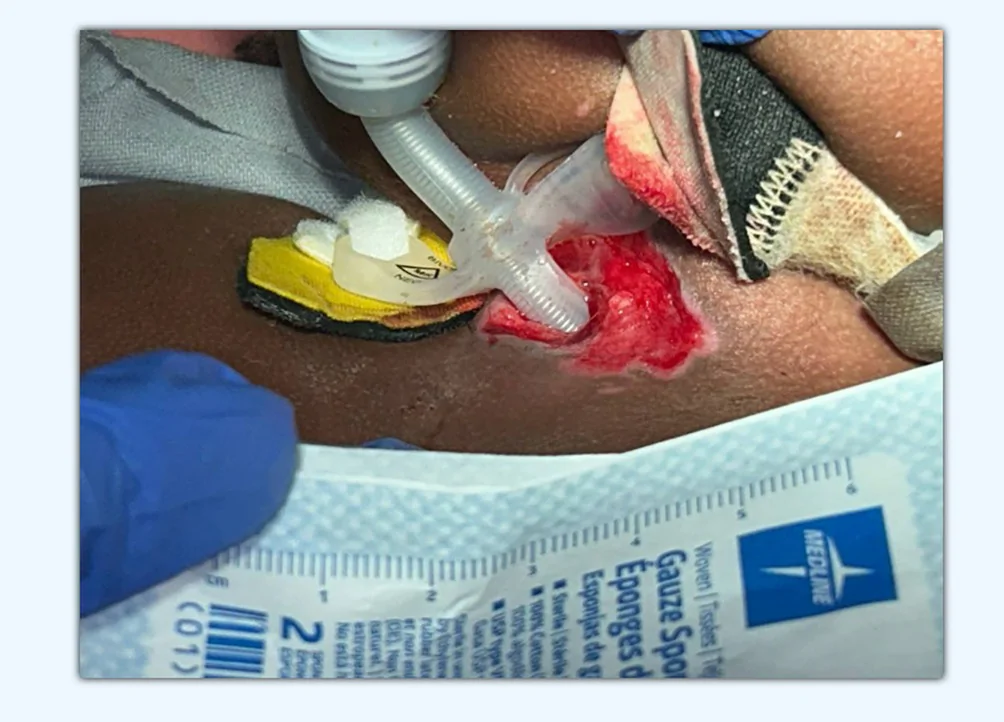
Surveillance microdirect laryngoscopy and bronchoscopy with significant supraglottic scarring, subglottic stenosis, and persistent edema. [Color figure can be viewed in the online issue, which is available at www.laryngoscope.com]

## 3 Discussion

Herein, we describe our experience with acute mucosal injury resulting in severe upper airway scarring in an infant with NS and severe HCM treated with MEK inhibitor, trametinib. Although MEK inhibitors show promise in reversing or stabilizing cardiac and lymphatic manifestations in NS, this case highlights a potential for significant mucosal toxicity, particularly in patients with preexisting airway vulnerability or prolonged intubation history.

NS is among the most prevalent RASopathies, characterized by dysregulation of the RAS/MAPK pathway, with resulting HCM present in approximately 20% of patients [1, 2, 3, 5]. Traditional management of NS‐associated HCM is largely supportive, and outcomes remain poor in high‐risk subgroups. Consequently, targeted therapies such as MEK inhibitors have gained attention as potential disease‐modifying interventions [4].

A recent systematic review by Gazzini et al. identified 23 pediatric patients with NS treated with MEK inhibitors—most commonly trametinib—for cardiac manifestations. Although most experienced clinical and echocardiographic improvement, nearly 50% had adverse events, and two discontinued therapy due to toxicity. Importantly, in several patients, discontinuation of therapy was followed by worsening of cardiac hypertrophy [4]. This underscores the disease‐modifying‐rather than curative‐nature of MEK inhibition and highlights the clinical challenge of weighing drug‐related toxicity against the risk of cardiac deterioration.

While prolonged intubation, tracheal compression, infection, and surgical intervention likely contributed to airway injury, several findings raised concern that these factors alone did not fully explain the severity of mucosal damage observed. Injury extended beyond areas typically associated with endotracheal tube trauma and involved the oropharynx, supraglottis, trachea, and bilateral mainstem bronchi, with diffuse mucosal sloughing, bleeding, and marked tissue fragility. In addition, the patient developed severe peristomal skin breakdown disproportionate to the expected postoperative course. The temporal association with trametinib escalation and interval stabilization following drug discontinuation further supported concern for trametinib‐associated mucosal toxicity. Pediatric‐specific dosing and toxicity monitoring guidelines for MEK inhibitors in RASopathies are lacking, with current protocols adapted from adult oncology. Given the variability in reported adverse events and our patient's airway complications, vigilant monitoring is warranted.

## 4 Conclusion

This case highlights a potentially underrecognized complication of trametinib therapy in infants with NS and severe HCM. Clinicians should maintain vigilance for airway toxicity, particularly in patients with existing respiratory vulnerability. Pediatric‐specific monitoring guidelines are needed to optimize safety while preserving the potential benefits of MEK inhibition.

## Funding

The authors have nothing to report.

## Ethics Statement

The authors have nothing to report.

## Conflicts of Interest

The authors declare no conflicts of interest.

## Data Availability

The data that support the findings of this study are available from the corresponding author upon reasonable request.
